# Apatinib suppresses tumor progression and enhances cisplatin sensitivity in esophageal cancer via the Akt/β-catenin pathway

**DOI:** 10.1186/s12935-020-01290-z

**Published:** 2020-05-27

**Authors:** Bin Wei, Yuanyuan Wang, Jiawei Wang, Xiaomin Cai, Lingyan Xu, Jingjing Wu, Ying Wang, Wen Liu, Yanhong Gu, Wenjie Guo, Qiang Xu

**Affiliations:** 1 Department of Oncology, The First Affiliated Hospital with Nanjing Medical University, 300 Guangzhou Road, Nanjing, 210029 China; 2 Department of Oncology, The Affiliated Huaian No.1 People’s Hospital of Nanjing Medical University, Huai’an, China; 3 State Key Laboratory of Pharmaceutical Biotechnology, School of Life Sciences, Nanjing University, 22 Hankou Road, Nanjing, 210093 China

**Keywords:** Apatinib, Esophageal cancer, Tumor progression, Cisplatin sensitivity, VEGFR2, Akt/β-catenin pathway

## Abstract

**Background:**

Esophageal cancer is the sixth leading cause of cancer-related mortality worldwide, which is partially due to limited progress of therapy. Apatinib, an inhibitor of VEGFR2, has a promising antitumor effect on malignancies. However, the underlying mechanism of its antitumor effect on esophageal cancer remains poorly understood.

**Materials and methods:**

Eighteen pairs of frozen esophageal cancer and their para-cancer samples and 25 paraffin specimens from advanced esophageal cancer patients treated with cisplatin-based regimen were collected. The effects of apatinib on cell growth, cell apoptosis, cell cycle and invasion/migration of esophageal cancer cells were assessed. Bioinformatics, luciferase reporter, immunoprecipitation and immunofluorescence assays were conducted for mechanic investigation. Quantitative RT-PCR, western blotting and immunohistochemistry were used to measure the expression of functional genes. Xenograft tumor growth of mice was performed.

**Results:**

We found that VEGFR2 was highly expressed in esophageal cancer and associated with poor efficacy of cisplatin-based treatment. Apatinib displayed profound actions against tumor cell growth of human esophageal cancer via promoting cell apoptosis and cell cycle arrest. Also, apatinib displayed the inhibitory effects on cell migration and invasion. Moreover, apatinib strongly suppressed the growth of esophageal cancer xenografts in mice. The effects of apatinib on esophageal cancer were partially dependent on its block of the VEGFR2/Akt/β-catenin pathway. Specifically, apatinib induced the degradation of β-catenin and decreased its transcriptional activity through Akt/GSK-3β repression. Further in vitro and in vivo studies revealed that low dose apatinib had a synergistic antitumor effect with cisplatin on esophageal cancer.

**Conclusion:**

Our study indicates that apatinib suppresses tumor progression and enhances cisplatin sensitivity in esophageal cancer by deactivating the Akt/β-catenin pathway. These findings provide a theoretical foundation for using apatinib as an effective therapeutic drug for esophageal cancer.

## Background

Esophageal cancer is the ninth most common malignancy with rapidly increasing incidence and the sixth leading cause of cancer-related death worldwide [1]. Due to the highly malignant nature and rapid development of esophageal cancer, as well as the poor therapeutic effect and high rate of recurrence and metastasis, the overall 5-year survival rate of esophageal cancer ranges from only 15% to 25% [2]. To date, no important progress has been made in the treatment of esophageal cancer, and the main treatments are still surgery, radiotherapy and chemotherapy. Therefore, studies on clinical trials of new drugs that could provide more effective therapy have attracted increasing attention.

Apatinib is a small-molecule multitargeted tyrosine kinase inhibitor that selectively inhibits the VEGFR-2, RET, c-Kit, and c-Src tyrosine kinases [3]. Increasing evidence suggests that apatinib exerts its promising antineoplastic activities by inhibiting the cell cycle, inducing apoptosis, suppressing angiogenesis, and inhibiting metastasis of cancer cells in a variety of tumors, such as colon cancer, thyroid cancer, liver cancer, cholangiocarcinoma and osteosarcoma [4–8]. With the tolerable side effects and improved survival benefits, apatinib has been approved by the China Food and Drug Administration for advanced gastric cancer in third-line or later treatment [9]. At present, a series of clinical trials of apatinib have been conducted and achieved improved clinical efficacy in multiple cancers, such as digestive tract cancer, breast cancer and lung cancer [10–15]. Few clinical retrospective studies have preliminarily suggested that apatinib alone or in combination with chemotherapy is efficacious for advanced esophageal cancer as a second-line or later treatment [16, 17]. However, the possible function and mechanism of apatinib in the treatment of esophageal cancer is still poorly understood.

In this study, we found that apatinib inhibited the growth of esophageal cancer and sensitized tumors to cisplatin by deactivating the Akt/β-catenin pathway, which provided a theoretical basis of apatinib as a potential candidate for the treatment of esophageal cancer.

## Materials and methods

### Patients collection

All patients with histological confirmed esophageal cancer were obtained from The Affiliated Huaian No.1 People’s Hospital of Nanjing Medical University (Huai’an, Jiangsu Province, China). A total of 18 pairs of resected fresh tumor and adjacent nontumor tissues were collected from the patients with stage I–III at the time of surgical resection. For this cohort, the median age was 60 (45–72 years) and 12 cases were male. Also, we obtained 25 advanced or metastatic cases (stage III or IV) treated with first-line cisplatin-based regimen, and their median age was 61 (48–81 years) and 16 cases were male. Treatment outcome was evaluated according to the Response Evaluation Criteria Evaluation in Solid Tumors (RECIST) system [18]. All patients signed their informed consent. The study protocol was approved by Human Ethics Committee of The Affiliated Huaian No.1 People’s Hospital of Nanjing Medical University.

### Cell lines

Human esophageal cancer cell lines KYSE30 and TE1 were purchased from Shanghai Cell Bank of Chinese Academy of Sciences (Shanghai, China). Mouse esophageal cancer AKR cell line was purchased from BeNa Culture collection of China (Suzhou, Jiangsu, China) [19]. These cells were cultivated in DMEM high-glucose medium (Gibco, USA) supplemented with 10% fetal bovine serum (Gibco, USA) and cultured in a humidified incubator with 5% CO_2_ at 37 °C.

### Chemicals and antibodies

Apatinib mesylate was donated by Hengrui Medicine Company (Lianyungang, Jiangsu, China). SC79, MG132 and cisplatin were purchased from Selleck Chemicals (Houston, TX, USA). For immunoblotting, the following primary antibodies were used: Bax (catalog no. 5023, 1:1000), Survivin (catalog no. 2808, 1:1000), p21 (catalog no. 2946, 1:1000), Cyclin D1 (catalog no. 2978, 1:1000), E-cadherin (catalog no. 3195, 1:1000), Vimentin (catalog no.5741, 1:1000), N-cadherin (catalog no. 14215, 1:1000), VEGFR2 (catalog no. 9698, 1:800), p-VEGFR2 (catalog no. 3770, 1:1000), β-catenin (catalog no. 8480, 1:1000 or 1:100), GSK-3β (catalog no. 9832, 1:2000), p-GSK-3β (catalog no. 5558, 1:1000), Lamin B1 (catalog no. 13435, 1:800) (Cell Signaling, Beverly, MA, USA), PCNA (catalog no. 60097-1-Ig, 1:500), Akt (catalog no. 10176-2-AP, 1:2000), p-Akt (catalog no. 66444-1-Ig, 1:1000) (Proteintech, Wuhan, China), CD31 (catalog no. sc-376764, 1:100), Ub (catalog no. sc-8017, 1:1000) (Santa, Dallas, Texas, USA) and β-Actin (catalog no. P30002, 1:5000), Goat Anti-Mouse IgG-HRP (catalog no. M21001, 1:2000), Goat Anti-Rabbit IgG-HRP (catalog no. M21002, 1:2000) (Abmart, Shanghai, China). Alexa Fluor 488 goat anti-rabbit/mouse IgG (catalog no. A-21222/A-10684, 1:500) was purchased from Thermo Fisher Scientific (MA, USA).

### In vitro cytotoxicity

The in vitro cytotoxicity was measured by Cell Counting Kit-8 (CCK-8) (Vicmed, Xuzhou, Jiangsu, China). Cells were plated in 96-well plates and treated with apatinib or cisplatin at indicated concentrations or time. Then, CCK-8 solution was added into each well and incubated at 37 °C for 2 h. The absorbance (450 nm) was measured in microplate reader (Bio-Tek Instruments, USA).

### Colony forming assay

Cells were placed into 6-well plates and treated with appropriate drug conditions, with medium replacement every 3–4 days. After 2 weeks, cells were fixed with 4% paraformaldehyde and stained with 0.1% crystal violet. The colonies were photographed with a digital camera and visible colonies were manually counted.

### Cell apoptotic analysis

The Annexin V-FITC/PI Apoptosis Detection Kit (KeyGEN, Nanjing, Jiangsu, China) was used to detect apoptotic cells. Cells were collected and mixed with Annexin V-FITC/PI buffer. Flow cytometry (Beckman, Brea, California, USA) was used to identify cells of normal status, early apoptosis, late apoptosis and death. The relative ratio of cells in apoptosis stages was analyzed [20].

### Cell cycle analysis

The cells were subjected to propidium iodide (PI) staining using the Cell Cycle Detection Kit (KeyGEN, Nanjing, Jiangsu, China) followed by flow cytometry. The levels of cells at different mitotic stages were analyzed by the ModFit program, version 2.0 (Becton–Dickinson, Franklin Lakes, New Jersey, USA) [21].

### Wound healing assay

Wound healing assay was performed by using a 24-well plate. After cells grew to 90% confluence gently remove the culture, and a linear wound was made by scrapping a pipet tip across the confluent cell layer. Cells were washed twice to remove detached cells. The remaining cells were incubated with appropriate apatinib conditions. All wounds were photographed using 200-fold magnification by a light microscope (Olympus, Lake Success, NY, USA). Then, the size of wound was observed and measured at the indicated times.

### Cell invasion assay

Cell invasion assay was performed using trans-well inserts with Matrigel (BD Biosciences, San Jose, CA, USA). Cells in serum-free medium were re-suspended in upper chambers and treated with apatinib. The cells were allowed to invade at 37 °C for 36 h toward a lower chamber filled with medium containing 20% fetal bovine serum. The cells that passed through the membrane were fixed with 4% paraformaldehyde and stained with 0.1% crystal violet. All specimens were photographed using 200-fold magnification by a light microscope (Olympus, Lake Success, NY, USA). The invaded cells were counted under an inverted microscope in five random fields.

### Quantitative real-time polymerase chain reaction (qPCR)

Total RNA was extracted with Trizol™ reagent (TaKaRa, Japan) and reverse transcribed with FastQuant RT Kit (Tiangen, Beijing, China) according to manufacturer’s instructions. Then, qPCR analysis was performed using the SYBR Green Kit (Invitrogen, Carlsbad, USA) and quantified by the Real-Time PCR Detection system (Roche, California, USA). Oligonucleotide primers were designed using Primer Bank (http://pga.mgh.harvard.edu/primerbank/index.html). Primer sequences were shown in Additional file 1: Table S1. Each sample was detected in triplicate and relative mRNA levels normalized to the expression of β-Actin were calculated using the 2^−ΔΔCt^ method.

### Western blotting

Whole cells were lysed in RIPA buffer (Beyotime, Shanghai, China) containing protease inhibitor cocktail (Vicmed, Xuzhou, Jiangsu, China) on ice. Equivalent loading protein from different samples was separated by 6–10% SDS–polyacrylamide gel electrophoresis (PAGE) and transferred onto polyvinylidene difluoride (PVDF) membrane (Millipore, MA, USA). Next, the membranes after blocking were incubated with different primary antibodies at 4 °C overnight. Finally, immunoreactive bands after incubation with secondary antibodies conjugated to peroxidase were detected using an ECL kit (Beyotime, Nangjing, Jiangsu, China) according to the manufacturer’s instructions.

### Bioinformatics analysis

The Kyoto Encyclopedia of Genes and Genomes (KEGG) network database was used to identify the genes enrichment in β-catenin pathway [22]. Then, UALCAN, an interactive web resource for analyzing transcriptome data from The Cancer Genome Atlas (TCGA) database, was employed to verify the expression level of enriched genes in esophageal cancer which were visualized with a heatmap [23].

### TCF-LEF reporter assay

β-catenin activity was examined using luciferase reporter assay of Cignal Lenti TCF/LEF Reporter (Qiagen, Venlo, Netherlands). After treatment, the reporter activity was quantified and standardized by using the ONE-Glo™ Luciferase Assay System (Promega, Madison, WI, USA) according to manufacturer’s protocol. The luciferase activity was quantified and standardized as described previously [24].

### Isolation of nuclear and cytoplasmic compartments

The nuclear and cytoplasmic compartment proteins of cells were isolated using a Nuclear and Cytoplasmic Protein Extraction Kit (KeyGEN Biotech, Jiangsu, China) according to the manufacturer’s instruction.

### Immunoprecipitation

Cells with different treatments were lysed by RIPA buffer and then anti-β-catenin-conjugated Protein A/G agarose (Santa Cruz, Santa, Dallas, Texas, USA) was used to incubate with cellular extracts at 4 °C overnight. Thereafter, the resultants were washed five times with cold RIPA buffer, boiled in SDS loading buffer, separated by SDS-PAGE followed by immunoblotting.

### Immunofluorescence

Cells treated with or without apatinib on coverslips were fixed in 4% formaldehyde, permeabilized with 0.5% Triton X-100 (PBS-T) and blocked with 5% BSA. Samples were then stained with anti-β-catenin, CD31 and Fluor-conjugated secondary antibody. The coverslips were counterstained with DAPI and covered with anti-fade reagent (Beyotime, Shanghai, China). Images were captured using 400-fold magnification with a confocal laser scanning microscope (Olympus, Lake Success, NY, USA).

### Animal experiment

Six- to eight-week-old female C57BL/6 mice were purchased from Model Animal Genetics Research Center of Nanjing University (Nanjing, China). Animal welfare and experimental procedures were carried out strictly following the Guide for the Care and Use of Laboratory Animals. All efforts were made to minimize animals’ suffering and to reduce the number of animals used. AKR cells (2.5 × 10^6^ cells in 100 μl PBS per mouse) were injected into mice by subcutaneous injection [25]. After 10 days, the animals were randomized (n = 5) and gavaged with apatinib (10 and 30 mg/kg/day) or an equal volume of 0.5% carboxymethylcellulose (CMC) as control. Cisplatin (1 mg/kg/day) was intraperitoneally injected with saline as the control. Tumor dimensions were measured using calipers, and tumor volumes were calculated using the following formula: (shortest diameter)^2^ × (longest diameter) × 0.5 [20]. After continuous observation, the tumor tissues were separated from sacrificed mice.

### Histologic analysis, TUNEL assay and immunohistochemistry (IHC)

TUNEL assay was performed using In Situ Cell Death Detection Kit (Roche, Basel, Switzerland). The immunostaining of PCNA was performed using a Real Envision Detection kit from the Gene Tech Company (Shanghai, China) according to the manufacturer’s instructions. All stained specimens were photographed using 200-fold magnification by a light microscope (Olympus, Lake Success, NY, USA).

### Statistical analysis

Statistical analyses were performed using the SPSS Statistics software (version 19.0, Chicago, USA). All experimental data were presented as the mean ± standard error of the mean (SEM). One-way ANOVA with Tukey’s correction was used to analyze statistically significant differences between multiple-group comparisons. A two-tailed Student’s t test was used to analyze statistically significant differences between two groups. *P* < 0.05 was considered statistically significant.

## Results

### VEGFR2 expression was higher and correlated with cisplatin-based treatment in esophageal cancer

**Fig. 1:**
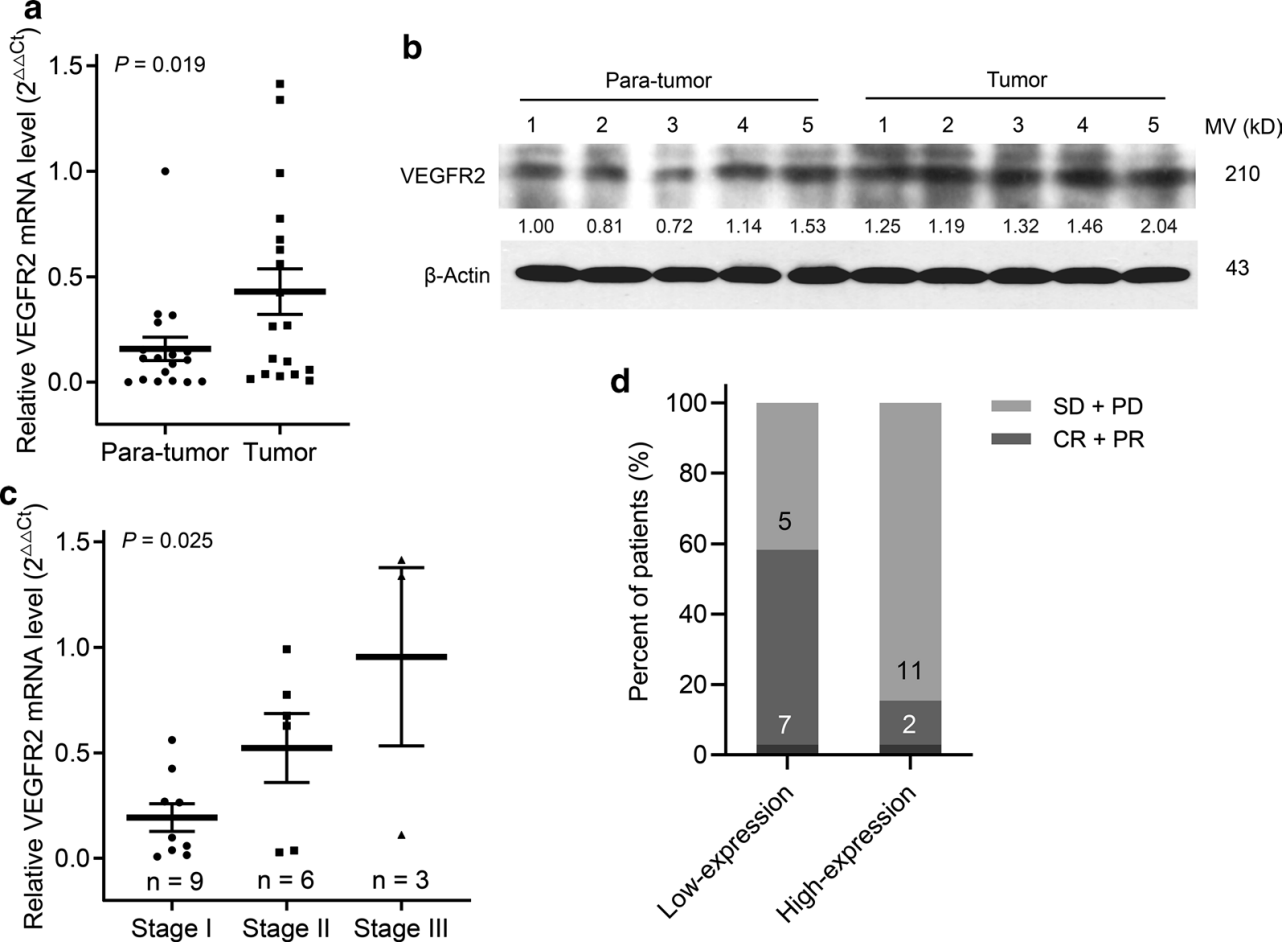
VEGFR2 expression was elevated in esophageal cancer and associated with the clinical outcome of cisplatin-based treatment. **a** qPCR analysis of VEGFR2 mRNA expression in 18 paired ESCC tissues and their adjacent non-tumor tissues. VEGFR2 levels were normalized to β-Actin expression. *P* value was determined by paired t test. **b** The VEGFR2 protein level in randomly matched tissues of 5 cases was determined by western blotting. β-Actin was used as a control. **c** Correlation of VEGFR2 mRNA expression with TNM stage. *P* value was determined by Student’s t-test. **d** Correlation of VEGFR2 mRNA expression and clinical outcome in 25 advanced esophageal cancer patients who underwent cisplatin-based treatment. CR, complete response; PR, partial response; SD, stable disease; PD, progressive disease. *P* value was determined by Chi-squared test

To determine the potential role of VEGFR2 in esophageal cancer, we evaluated the VEGFR2 expression in 17 paired cancer and para-cancer tissues. VEGFR2 mRNA and protein expressions were markedly increased in cancer tissues compared with para-carcinoma tissues (Fig. 1a, b). Also, the higher VEGFR2 mRNA expression was associated with a greater TNM stage (Fig. 1c). Additionally, we also explored the relationship between VEGFR2 mRNA expression and chemotherapy sensitivity of cisplatin-based regimen in 25 patients with advanced esophageal cancer. The patients were divided into two groups using median value as the cut-off point, and patients with low VEGFR2 mRNA expression had a high objective response rate (58.3 vs. 15.4%, *P *= 0.025; Fig. 1d) compared with those with high expression.

### Apatinib suppressed cell proliferation via inducing cell apoptosis and cell cycle arrest in esophageal cancer

**Fig. 2:**
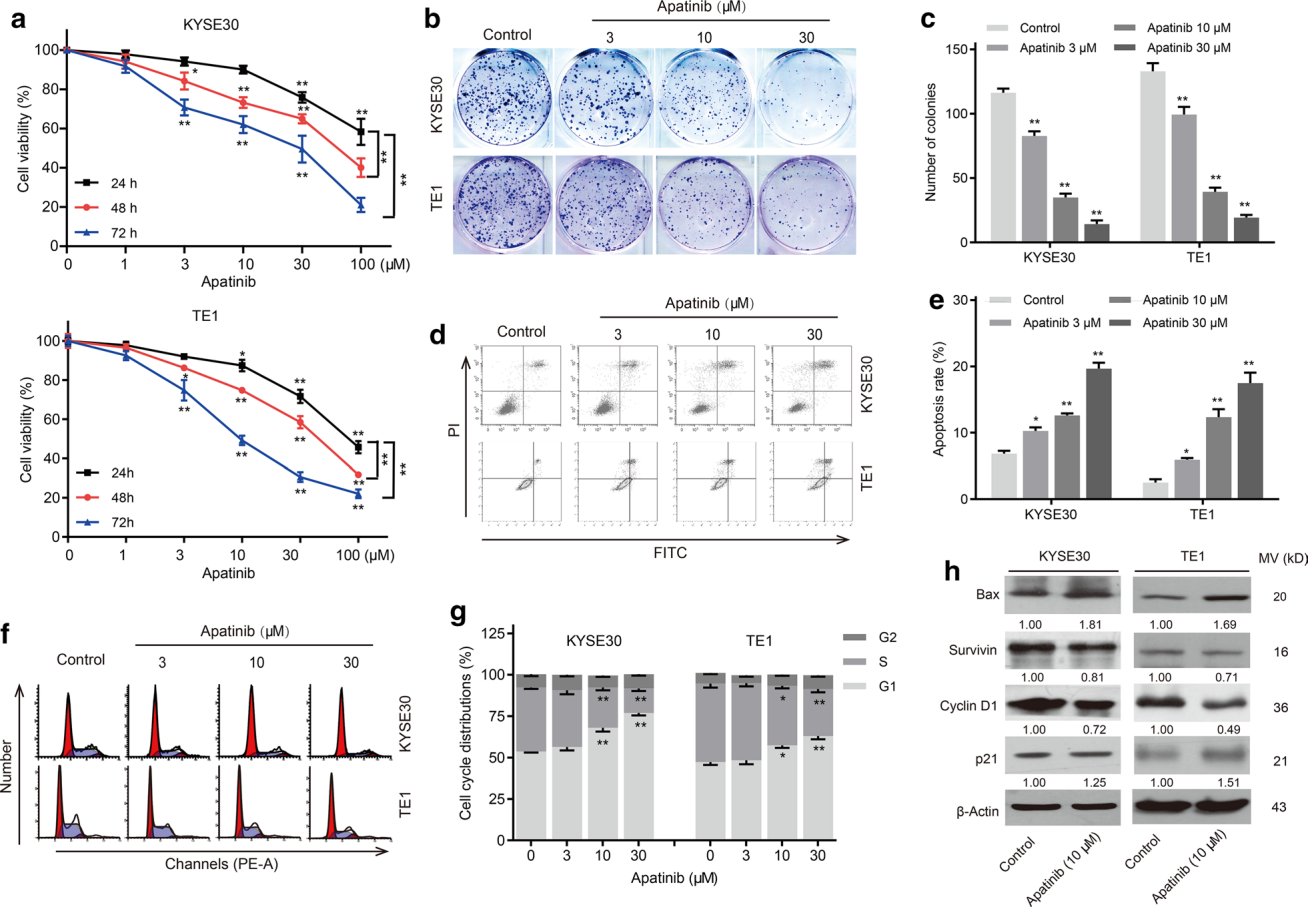
Apatinib inhibited esophageal cancer cell growth via promoting cell apoptosis and suppressing cell cycle progression in vitro. **a** Cell viability of KYSE30 and TE1 cell lines detected by a CCK-8 kit after apatinib treatment (1, 3, 10, 30 and 100 μM) for the indicated time. *P* values were determined by one-way ANOVA with Tukey’s correction. *P < 0.05, **P < 0.01. **b** Colony formation ability of KYSE30 and TE1 cells was inhibited by apatinib (3, 10 and 30 μM). **c** Quantitative analysis of the number of colonies. *P* values were determined by one-way ANOVA with Tukey’s correction. *P < 0.05, **P < 0.01. **d** KYSE30 and TE1 cells were treated with apatinib (3, 10 and 30 μM) for 36 h. Apoptosis was determined by flow cytometry. **e** Quantitative analysis of the apoptotic rate. *P* values were determined by one-way ANOVA with Tukey’s correction. *P < 0.05, **P < 0.01. **f** KYSE30 and TE1 cells were treated with apatinib (3, 10 and 30 μM) for 36 h. The number of cells in different stages of the cell cycle was determined by flow cytometry. **g** Quantitative analysis of cell cycle distribution. *P* values were determined by one-way ANOVA with Tukey’s correction. *P < 0.05, **P < 0.01. **h** KYSE30 and TE1 cells were treated with apatinib (10 μM) for 24 h. The protein levels of Survivin, Bax, Cyclin D1 and p21 were determined by western blotting. Data are representative of three independent experiments (mean and SEM in **a**, **c**, **e**, **g**)

The cytostatic action of apatinib on esophageal cancer cell lines (KYSE30 and TE1) was assessed by CCK-8 assay. The cell viability of the two cell lines decreased with increasing concentrations and exposure time, indicating that apatinib suppressed cell proliferation in a dose- and time-dependent manner (Fig. 2a). Moreover, a colony formation assay revealed that the number and size of colonies formed by the two tumor cell lines were significantly inhibited by apatinib in a dose-dependent manner (Fig. 2b, c).

Furtherly, flow cytometric analysis of KYSE30 and TE1 cells treated with apatinib showed significant apoptosis induction when compared to that of the control (Fig. 2d, e). Meanwhile, we discovered that apatinib significantly arrested both cell lines at the G0/G1 phase, but fewer cells were in the S phase (Fig. 2f, g). The results from the western blotting assay revealed that apatinib altered the expression of cell cycle- and apoptosis-associated proteins, including upregulation of Bax and p21 and downregulation of Survivin and Cyclin D1 (Fig. 2h). These data indicated that apatinib exerted its antigrowth effect by inducing cell apoptosis and blocking cell cycle progression in esophageal cancer cells.

### Apatinib inhibited cell migration and invasion of esophageal cancer

**Fig. 3:**
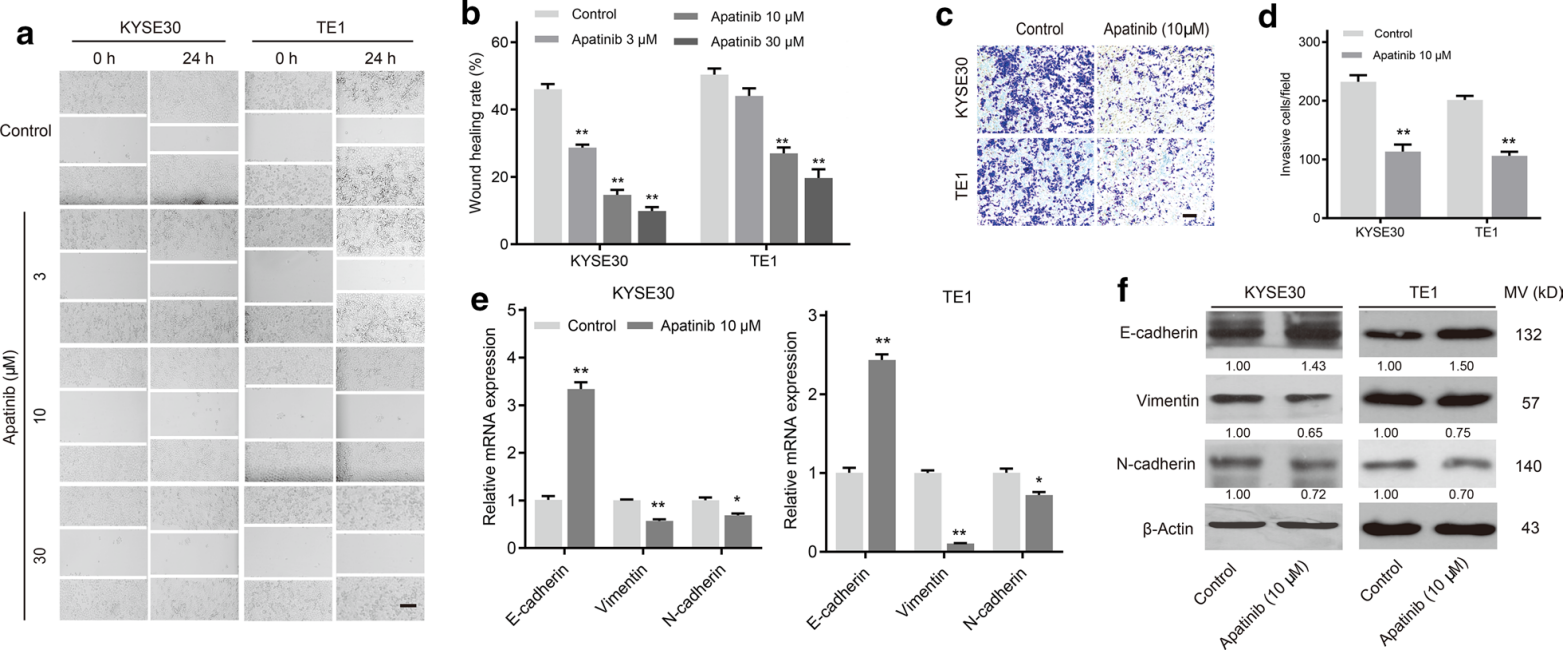
Apatinib suppressed the migration and invasion of esophageal cancer cells in vitro. **a** KYSE30 and TE1 cells treated with apatinib (3, 10 and 30 μM) for 24 h were scraped and imaged immediately (0 h) and later (24 h), and images of the wound gap were taken. Scale bar, 200 μm. **b** Quantitative analysis of the wound healing rate. *P* values were determined by one-way ANOVA with Tukey’s correction. *P < 0.05, **P < 0.01. **c** After apatinib (10 μM) treatment for 36 h, invaded cells were stained and counted using microscopy. Scale bar, 50 μm. **d** Quantitative analysis of the number of invaded cells. *P* values were determined by Student’s t test. *P < 0.05. KYSE30 and TE1 cells were treated with apatinib (10 μM) for 24 h. **e** The mRNA expression levels of E-cadherin, Vimentin and N-cadherin were determined by qPCR. *P* values were determined by Student’s t test. *P < 0.05, **P < 0.01. **f** The protein levels of E-Cadherin, Vimentin and N-cadherin were determined by western blotting. Data are representative of three independent experiments (mean and SEM in **b**, **d**, **e**)

To investigate whether apatinib could inhibit the cellular motility of esophageal cancer, wound healing and transwell assays were conducted. The migration speed of KYSE30 and TE1 cells was significantly decreased following exposure to apatinib relative to that of the control group (Fig. 3a, b). Furthermore, results of another transwell assay showed that apatinib markedly suppressed the invasion ability of these two cell lines (Fig. 3c, d).

Epithelial–mesenchymal transition (EMT) plays a critical role during tumor metastasis, and the expression of markers related to this process was examined by qPCR and western blotting. We found that apatinib treatment resulted in the upregulation of the epithelial marker E-cadherin and the downregulation of mesenchymal markers, including Vimentin and N-cadherin, at both the mRNA and protein levels (Fig. 3e, f). These results suggested that apatinib might reverse the EMT process and control the cell metastasis of esophageal cancer.

### Apatinib regulated β-catenin signaling in esophageal cancer

**Fig. 4:**
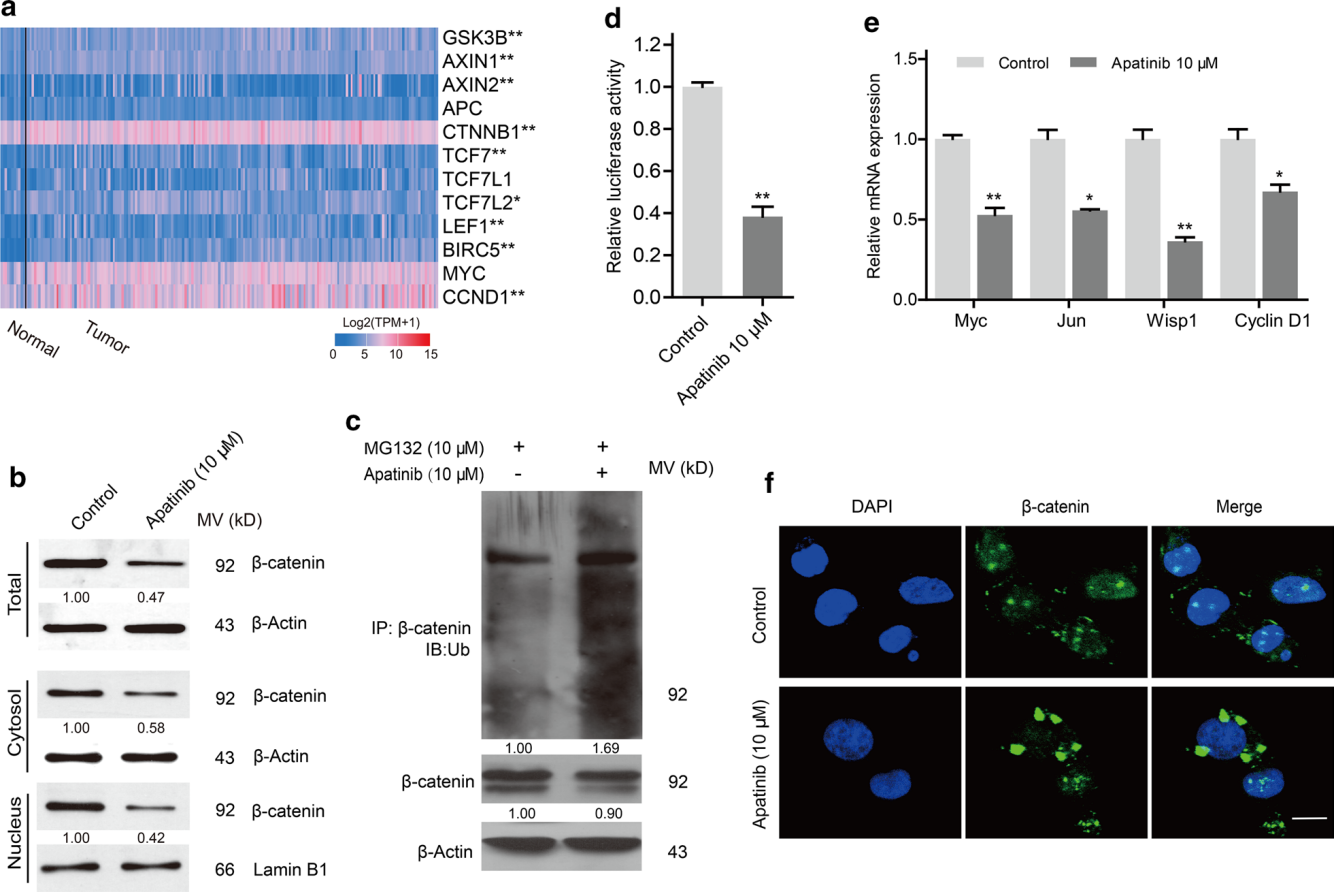
Apatinib negatively regulated the β-catenin signaling in esophageal cancer. **a** Heatmap for β-catenin signaling in esophageal cancer tissues compared to that in normal tissues using TCGA data. *P < 0.05, **P < 0.01. **b** KYSE30 cells were treated with apatinib (10 μM) for 24 h. Western blotting was performed to detect β-catenin expression in whole-cell lysates, as well as in the cytoplasmic and nuclear fractions. **c** KYSE30 cells were pretreated with MG132 (10 μM) and exposed to apatinib (10 μM) for 24 h. Whole cellular extracts were prepared for coimmunoprecipitation assay with anti-β-catenin followed by immunoblotting with anti-Ub antibody. KYSE30 cells were incubated with DMSO or apatinib (10 μM) for 24 h. **d** β-catenin-TCF/LEF transcriptional activity was detected by luciferase reporter assays. *P* values were determined by Student’s t test. **P < 0.01. **e** The mRNA expression levels of Myc, Jun, Wisp1 and Cyclin D1 were examined by qPCR. *P* values were determined by Student’s t test. *P < 0.05, **P < 0.01. **f** Representative images showing double immunofluorescence staining for β-catenin (green) and nuclei (DAPI, blue). Scale bar, 25 μm. Data are representative of three independent experiments (mean and SEM in **d**, **e**)

β-Catenin-mediated signaling regulates tumor initiation and progression in multiple malignancies [26]. Here, we analyzed the transcriptome data of esophageal cancer via the UALCAN data portal and found that the majority of genes in the pathway were differentially expressed in tumor tissues compared to normal tissues (Fig. 4a). Then, we investigated whether β-catenin and its downstream effectors were affected by apatinib treatment in the KYSE30 cell line. Decreased expression of β-catenin protein was observed in cells exposed to apatinib, and the expression trend was consistent with the cytoplasm and nucleus (Fig. 4b). To further clarify whether the decrease in β-catenin was mediated by the ubiquitin–proteasome degradation pathway, we used MG132 to inhibit the proteasome and found that ubiquitinated β-catenin was increased in the apatinib-treated group (Fig. 4c). Furthermore, a luciferase reporter assay discovered that apatinib attenuated β-catenin-mediated transcriptional activity (Fig. 4d). Additionally, the expression levels of several downstream genes of β-catenin were significantly decreased, including Myc, Jun, Wisp1 and Cyclin D1 (Fig. 4e). In addition, immunofluorescence assay was employed to investigate the location of β-catenin, and the results indicated that treatment with apatinib promoted the transfer of β-catenin from the nucleus to the cytoplasm in esophageal cancer cells (Fig. 4f). Overall, our data demonstrated that the antitumor effects of apatinib occurred through induction of β-catenin degradation.

### Apatinib suppressed β-catenin signaling via VEGFR2/Akt/GSK-3β

**Fig. 5:**
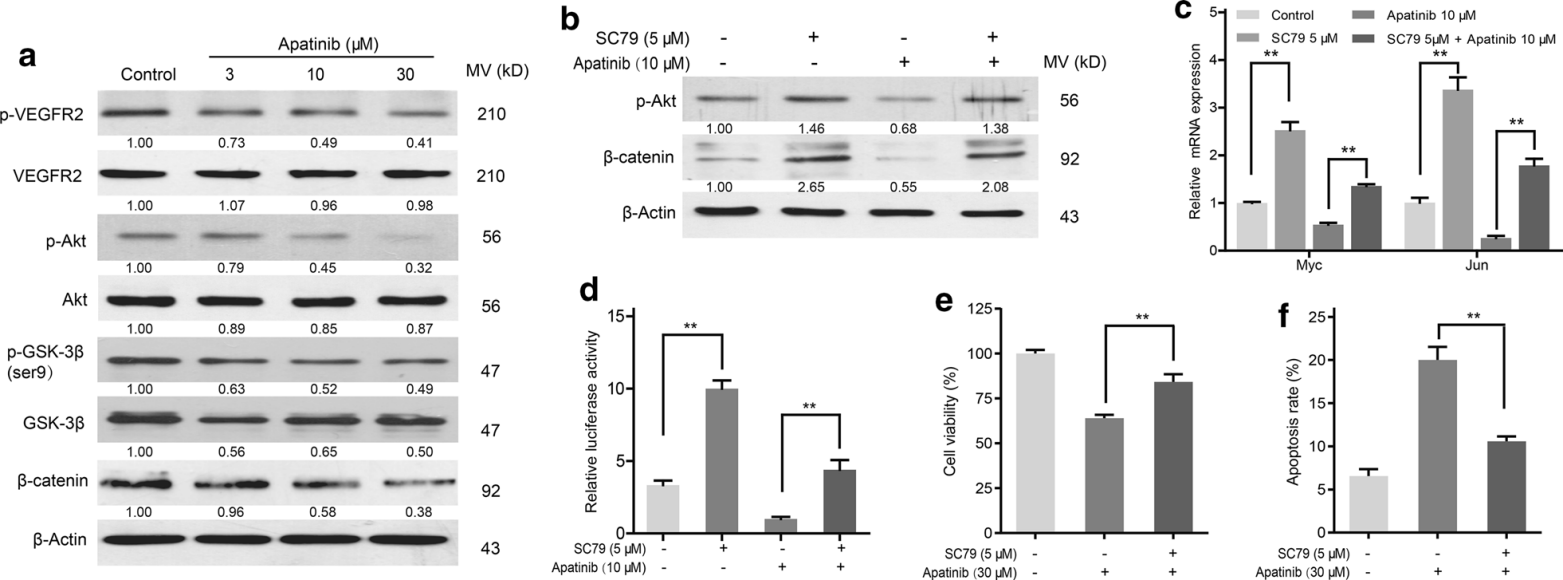
Apatinib suppressed the β-catenin pathway via VEGFR2/Akt/GSK-3β. **a** KYSE30 cells were exposed to apatinib (3, 10 and 30 μM) for 24 h. The protein levels of Akt, p-Akt, GSK-3β, p-GSK-3β and β-catenin were detected by western blotting. KYSE30 cells were pretreated with DMSO or SC79 (5 μM) and then exposed to apatinib (10 μM) for 24 h. **b** The protein levels of p-Akt and β-catenin were detected by western blotting. **c** The mRNA expression levels of Myc and Jun were examined by qPCR. *P* values were determined by Student’s t test. **P < 0.01. **d** The transcriptional activity was detected by luciferase reporter assays. *P* values were determined by Student’s t test. **P < 0.01. KYSE30 cells were pretreated with DMSO or SC79 (5 μM) and then exposed to apatinib (30 μM) for 24 h. **e** Cell viability of KYSE30 cell line detected by a CCK-8 kit. *P* values were determined by Student’s t test. **P < 0.01. **f** Apoptosis was determined by flow cytometry. *P* values were determined by Student’s t test. **P < 0.01. Data are representative of three independent experiments (mean and SEM in **c**–**f**)

Recent research has shown VEGFR2 mediates the Akt/GSK3β signaling [4]. Also, the Akt/GSK-3β pathway triggers a network that positively regulates tumor progression by activating β-catenin [27]. We found that the protein expression of p-VEGFR2, p-Akt, p-GSK-3β Ser9 and β-catenin was decreased in KYSE30 cells treated with apatinib (Fig. 5a). To confirm the effect of apatinib on the Akt/β-catenin pathway, cells were pretreated with the Akt agonist SC79 to alter its activity. We demonstrated a decrease in the protein expression of p-Akt and β-catenin (Fig. 5b), the mRNA expression of Myc and Wisp1 (Fig. 5c), and the transcriptional activity of apatinib were restored by SC79 treatment (Fig. 5d). Then, we examined the role of Akt in apatinib-mediated growth inhibition of esophageal cancer. The results showed that the apatinib-mediated proliferation inhibition and apoptosis induction was reversed by SC79 (Fig. 5e, f). Collectively, these data revealed a possible mechanism by which apatinib inhibited esophageal cancer progression by suppressing the VEGFR2/Akt/GSK-3β/β-catenin pathway.

### Apatinib inhibited tumor growth of esophageal cancer via the VEGFR2/Akt/β-catenin pathway in vivo

**Fig. 6:**
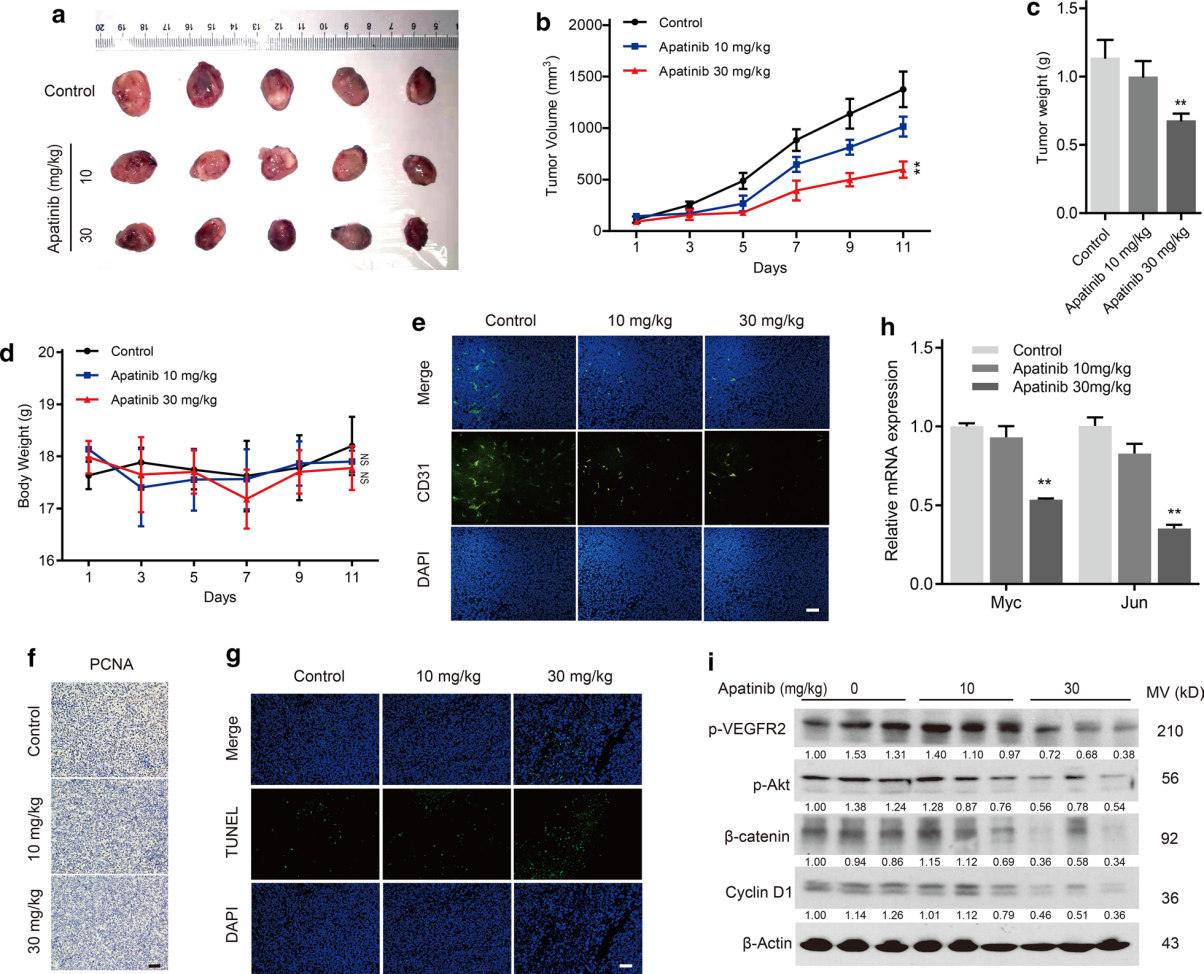
Apatinib inhibited esophageal cancer growth via the Akt/β-catenin pathway in vivo. **a** All xenograft models were sacrificed and photographed after treatment with vehicle or apatinib (10 and 30 mg/kg/day). n = 5 mice per group. **b** Tumor volume (expressed in mm^3^) of xenografts was monitored every 2 days. Day 1 represents the first day of apatinib treatment. *P* values were determined by one-way ANOVA with Tukey’s correction. **P < 0.01. **c** Tumors were weighed at the end of the experiment, and weight was expressed in gram. *P* values were determined by one-way ANOVA with Tukey’s correction. **P < 0.01. **d** The weight of the mouse was monitored every 2 days, and weight was expressed in gram. *P* values were determined by one-way ANOVA with Tukey’s correction. NS represents no significance. **e** Xenograft tumors in the indicated groups were detected by immunofluorescence. Representative images of CD31 (green) and nuclei staining (DAPI, blue). Scale bar, 100 μm. **f** PCNA staining in the indicated groups was detected by IHC. Scale bar, 100 μm. **g** Xenograft tumors in the indicated groups were detected by immunofluorescence. Representative images of TUNEL (green) and nuclei staining (DAPI, blue). Scale bar, 100 μm. **h** The mRNA expression levels of Myc and Jun in tumor samples from the indicated groups were examined by qPCR. Data represent experiments in three independent tumors. *P* values were determined by one-way ANOVA with Tukey’s correction. **P < 0.01. **i** The protein levels of p-VEGFR2, p-Akt, β-catenin and CyclinD1 in tumor samples from the indicated groups were determined by western blotting

To evaluate the antitumor effect of apatinib in vivo, xenograft mouse models were treated with apatinib by gavage. The growth of tumor xenografts and tumor weights were significantly inhibited by apatinib at a dose of 30 mg/kg (Fig. 6a–c). Also, no mice died during the experiment, and no significantly difference in bodyweight was observed among control and treatment groups (Fig. 6d). The blood vessels in tumors were detected using the CD31 staining, and we found apatinib treatment inhibited angiogenesis of esophageal cancer (Fig. 6e). Furthermore, apatinib inhibited the expression of PCNA (Fig. 6f). In contrast, TUNEL-positive cells were also increased in tumors that received apatinib treatment (Fig. 6g). In this case, dephosphorylation of VEGFR-2 and Akt, β-catenin degradation and decreased levels of Cyclin D1 in tumors were observed after apatinib treatment (Fig. 6i). Furthermore, the mRNA levels of Myc and Jun showed a downward trend (Fig. 6h). Taken together, these in vivo data indicated that apatinib could inhibit xenograft tumor growth in esophageal cancer by blocking the Akt/β-catenin pathway.

### Apatinib sensitized esophageal cancer to cisplatin via the Akt/β-catenin pathway

**Fig. 7:**
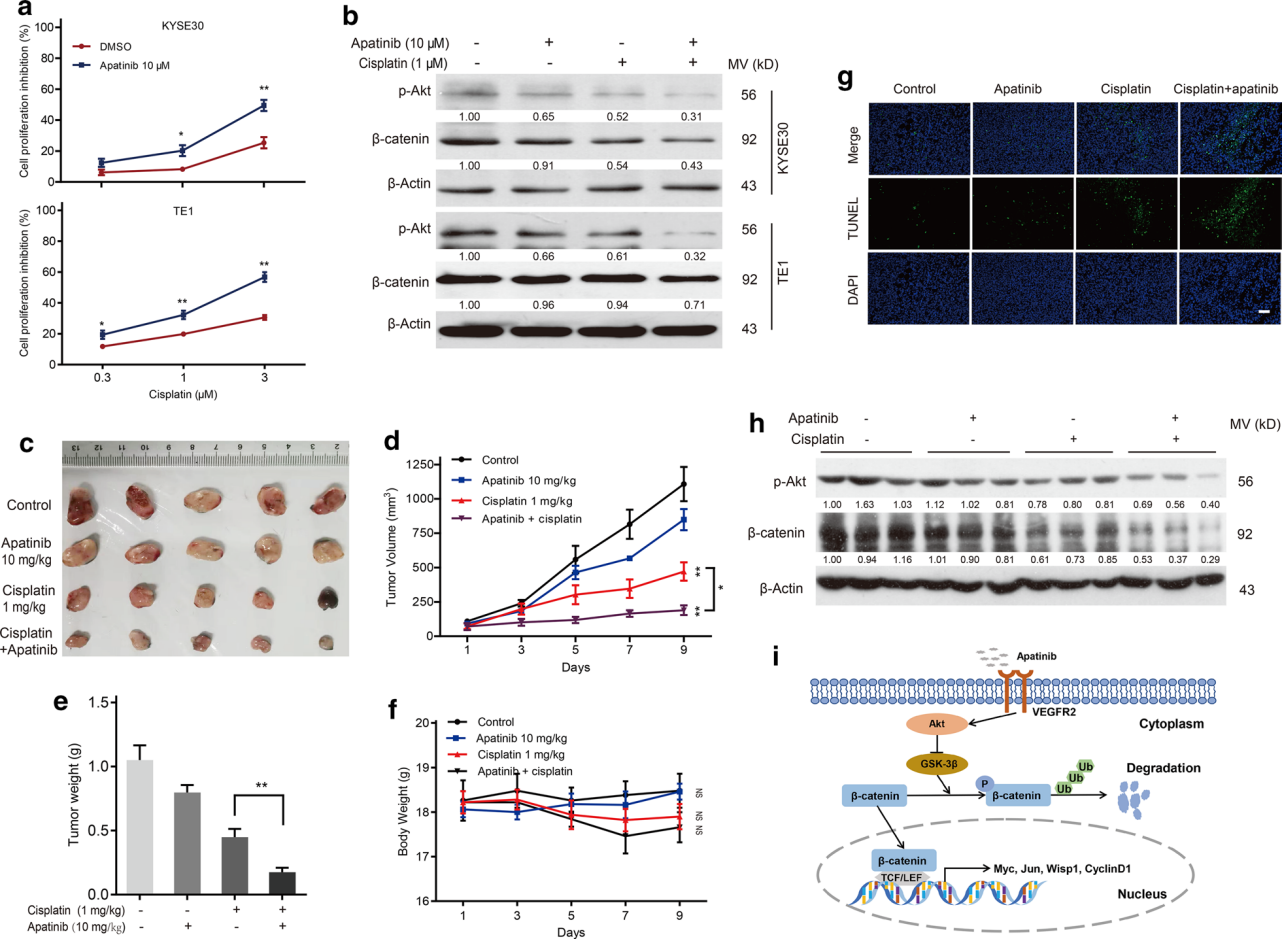
Apatinib sensitized esophageal cancer to cisplatin by inhibiting the Akt/β-catenin pathway. **a** Cell proliferation inhibition of KYSE30 and TE1 cells was detected by a CCK-8 kit after cisplatin (0.3, 1 and 3 μM) treatment with or without apatinib (10 μM) for 24 h. *P* values were determined by Student’s t test. Data are representative of three independent experiments (mean and SEM). *P < 0.05, **P < 0.01. **b** KYSE30 and TE1 cells were treated with apatinib (10 μM) or/or cisplatin (1 μM) for 24 h. The protein levels of p-Akt and β-catenin were detected by western blotting. **c** All xenograft models were sacrificed and photographed after treatment with vehicle, low-dose apatinib (10 mg/kg/day), cisplatin (1 mg/kg/day) or low-dose apatinib plus cisplatin. n = 5 mice per group. **d** Tumor volume (expressed in mm^3^) of xenografts was monitored every 2 days. Day 1 represents the first day of treatment. *P* values were determined by one-way ANOVA with Tukey’s correction. *P < 0.05, **P < 0.01. **e** Tumors were weighed at the end of the experiment, and weight was expressed in gram. *P* values were determined by one-way ANOVA with Tukey’s correction. **P < 0.01. **f** The weight of the mouse was monitored every 2 days, and weight was expressed in gram. *P* values were determined by one-way ANOVA with Tukey’s correction. NS represents no significance. **g** Xenograft tumors in the indicated groups were detected by immunofluorescence. Representative images of TUNEL (green) and nuclei staining (DAPI, blue). Scale bar, 100 μm. **h** The protein levels of p-Akt and β-catenin in tumor samples from the indicated groups were determined by western blotting. **i** Schematic diagram of the molecular mechanism by which apatinib regulates the biological functions of esophageal cancer

To explore whether apatinib could sensitize esophageal cancer to cisplatin, cotreatment with apatinib and cisplatin was performed both in vitro and in vivo. The CCK-8 assay showed that cisplatin-induced growth inhibition was increased in both KYSE30 and TE1 cells cotreated with apatinib (Fig. 7a). Additionally, we discovered the additive effects of two drugs on Akt/β-catenin signaling, and these effects led to lower p-Akt and β-catenin levels than those seen with either treatment alone (Fig. 7b). Furthermore, the in vivo efficacies of low-dose apatinib and/or cisplatin were investigated in the xenograft models. The tumor volumes in mice receiving cotreatment with apatinib and cisplatin were significantly smaller than those in the cisplatin only group (Fig. 7c–e). More importantly, combination of apatinib and cisplatin has no an obvious effect on bodyweight compared with the cisplatin only group (Fig. 7f). Compared with cisplatin alone, TUNEL-positive cells were increased and expressions of p-Akt and β-catenin protein were decreased in tumors after cisplatin and apatinib treatment (Fig. 7g, h). These data indicated that apatinib enhanced the chemosensitivity of esophageal cancer to cisplatin via suppression of the Akt/β-catenin pathway.

## Discussion

Esophageal cancer is a common type of gastrointestinal malignancy with a high mortality rate in China [2, 28]. With the limited progress seen thus far in medical treatment, the prognosis of esophageal cancer has not been significantly improved [29]. Apatinib, as a highly selective tyrosine kinase inhibitor, has exerted promising antitumor effects on malignant tumors [13]. In the present study, our data demonstrated that apatinib inhibited tumor progression and promoted cisplatin sensitivity by negatively regulating the Akt/β-catenin pathway in esophageal cancer (Fig. 7i).

First, we found that VEGFR2 mRNA and protein expression were remarkably increased in esophageal cancer samples compared with associated non-tumor tissues. The finding was in agreement with the results reported in several human cancers [7, 30]. Also, our study confirmed that high VEGFR2 expression was associated with later TNM stage, which might suggest that VEGFR2 was essential for ESCC progression. Some studies have higher VEGFR2 expression was associated with worse overall survival of malignancies [31, 32]. Then, we revealed that apatinib (highly selective VEGFR2 antagonist) inhibited the growth of human esophageal cancer cells in a time- and dose-dependent manner. Consistent with previous studies in human colon cancer, osteosarcoma and thyroid cancer [4, 7, 8, 33], we also demonstrated that apatinib induced cell apoptosis and cycle arrest at G0/G1 phase in vitro. The results of the xenograft assay further showed that apatinib significantly blocked the tumor growth of esophageal cancer, as well as inhibited angiogenesis, promoted apoptosis and suppressed the cell cycle in vivo. In addition, our findings suggested that apatinib might inhibit the metastasis of esophageal cancer via EMT inhibition, which was in line with the findings of a study in osteosarcoma [6]. Therefore, we found that apatinib inhibited tumor progression by not only inhibiting angiogenesis but also suppressing tumor cell growth and metastasis.

The Wnt/β-catenin pathway has been implicated in tumorigenesis and progression in many cancer types [34]. Consistent with Deng’s results [35], TCGA data analysis verified that most β-catenin signaling-related genes were differentially expressed in esophageal cancer. Here, we discovered that the level of total or nuclear β-catenin was decreased in response to apatinib treatment. When the upstream pathways are suppressed, β-catenin is phosphorylated and then delivered to the proteasome for degradation [36]. Upon apatinib treatment, an increased level of ubiquitination of β-catenin was observed, which could be degraded by ubiquitination. β-catenin accumulates in the cytoplasm and transfers to the nucleus, where it interacts with TCF/LEF transcription factors, thus affecting a variety of biological processes, including the cell cycle, apoptosis, angiogenesis and metastasis [37, 38]. Consistent with the reduced amount of β-catenin seen in the nucleus, apatinib confined TCF/LEF-mediated transcriptional activity. Accordingly, apatinib blocked β-catenin signaling, which partially explained its antitumor effects.

Recently, studies have suggested that the antitumor function of apatinib may be achieved by regulating VEGFR2-mediated multimodality pathways, such as the RAF/ERK, MAPK, and STAT3/Bcl-2 pathways [5, 33, 39]. Besides, VEGFR2 can positively regulate Akt/GSK-3β signaling [4]. Among Wnt-independent pathways, the Akt can indirectly regulate β-catenin expression by phosphorylating GSK-3β at Ser9 to inactivate its kinase activity [27]. In our study, the degradation of β-catenin, which was mediated by apatinib, was accompanied by a decrease in p-VEGFR2, p-AKT and p-GSK-3β. Further rescue experiments discovered that the decreased expression level and transcriptional activity of β-catenin caused by apatinib were recovered with pretreatment with SC79 (an Akt agonist). Meanwhile, apatinib-mediated cell growth suppression was also reversed by SC79. Similarly, apatinib inhibited the phosphorylation of VEGFR2 or Akt and then increased instability of β-catenin in vivo. Based on our results and the results of others, we speculate that apatinib regulates Akt/β-catenin signaling through its VEGFR2 inactivation, and this mechanism requires further exploration.

Combination strategies that can improve clinical efficacy have become the trend in antitumor therapy, such as chemotherapy combined with targeted therapy or immunotherapy [40]. According to existing research results, VEGFR2 confers to chemoresistance in cancers [41–43]. In our study, we discovered that high VEGFR2 associated with poor clinical efficacy of cisplatin-based chemotherapy in advanced esophageal cancer. Existing clinical studies have shown that apatinib causes some adverse reactions, such as hypertension, hand and foot syndrome, proteinuria, diarrhea, and the incidence of adverse reactions are increased with the combination of regular dose of apatinib and chemotherapy [17, 44]. Data from a clinical study have demonstrated that low dose apatinib plus chemotherapeutic agent is effective without increasing adverse reactions in lung cancer [45]. Thus, we selected the low dose apatinib to explore its effects on cisplatin sensitivity in esophageal cancer. Here, we discovered that cotreatment with cisplatin and low dose apatinib more effectively inhibited cancer cell proliferation than cisplatin alone in vitro and in vivo. In addition, the combination suppressed the Akt/β-catenin pathway synergistically.

In conclusion, we demonstrated that apatinib inhibited the progression and enhanced the cisplatin sensitivity of esophageal cancer in vivo and in vitro. Moreover, suppression of VEGFR2/Akt/GSK-3β-mediated β-catenin stability and transcriptional activity was implicated in the antitumor effects of apatinib. These findings provide a theoretical foundation for using apatinib as an effective therapeutic strategy for esophageal cancer.

## Conclusion

In this study, we discovered that apatinib inhibited the growth of esophageal cancer and sensitized tumors to cisplatin by deactivating the Akt/β-catenin pathway, which provided a theoretical basis for apatinib as a potential therapeutic strategy for esophageal cancer.

## Supplementary information

**Additional file 1: Table S1.** Primers used for qPCR.

## Data Availability

The data that support the findings of this study are available from the corresponding author upon reasonable request.

## Abbreviations

PI: Propidium iodide
qPCR: Quantitative real-time polymerase chain reaction
PAGE: Polyacrylamide gel electrophoresis
PVDF: Polyvinylidene difluoride
KEGG: Kyoto encyclopedia of genes and genomes
TCGA: The cancer genome atlas
IHC: Immunohistochemistry
EMT: Epithelial–mesenchymal transition
