# Supplementary material for: Apatinib suppresses tumor progression and enhances cisplatin sensitivity in esophageal cancer via the Akt/β-catenin pathway
Source: Cancer Cell Int. 2020 May 27;20:198. doi: 10.1186/s12935-020-01290-z (PMC7254695; doi:10.1186/s12935-020-01290-z)
Supplement: Supplementary file 1 — Additional file 1: Table S1. Primers used for qPCR. [file 12935_2020_1290_MOESM1_ESM.docx]

**Table S1. Primers used for qPCR.**

| **Gene name** | **Sequence (5’-3’)** | |
| --- | --- | --- |
|  | **Forward** | **Reverse** |
| Human | | |
| β-actin | GCCAGAGGCGTACAGGGATA | GGCCCAATAATCAGAGTGGCA |
| VEGFR2 | GGCCCAATAATCAGAGTGGCA | CCAGTGTCATTTCCGATCACTTT |
| E-cadherin | GTCTCTCTCACCACCTCCACAG | CTCGGACACTTCCACTCTCTTT |
| Vimentin | GAAGAGAACTTTGCCGTTGAAG | GAAGGTGACGAGCCATTTC |
| N-cadherin | TGCTACTTTCCTTGCTTCTGAC | TAACACTTGAGGGGCATTGTC |
| Myc | GGCTCCTGGCAAAAGGTCA | CTGCGTAGTTGTGCTGATGT |
| Wisp1 | GTGCTGTAAGATGTGCGCTCA | CACTCCTATTGCGTACCTCGG |
| CyclinD1 | GCTGCGAAGTGGAAACCATC | CCTCCTTCTGCACACATTTGAA |
| Jun | TCCAAGTGCCGAAAAAGGAAG | CGAGTTCTGAGCTTTCAAGGT |
| Mouse | | |
| β-actin | GGCTGTATTCCCCTCCATCG | CCAGTTGGTAACAATGCCATGT |
| Myc | ATGCCCCTCAACGTGAACTTC | CGCAACATAGGATGGAGAGCA |
| Jun | CCTTCTACGACGATGCCCTC | GGTTCAAGGTCATGCTCTGTTT |
